# Supplementary material for: Antitumor Activity by an Anti-CD44 Variant 9 Monoclonal Antibody in Gastric and Colorectal Cancer Xenograft Models
Source: Int J Mol Sci. 2025 Sep 19;26(18):9170. doi: 10.3390/ijms26189170 (PMC12470293; doi:10.3390/ijms26189170)
Supplement: Supplementary file 1 [file ijms-26-09170-s001.zip › Supplementary Figure S1, S2 and S3.pdf]

International Journal of
Molecular Sciences

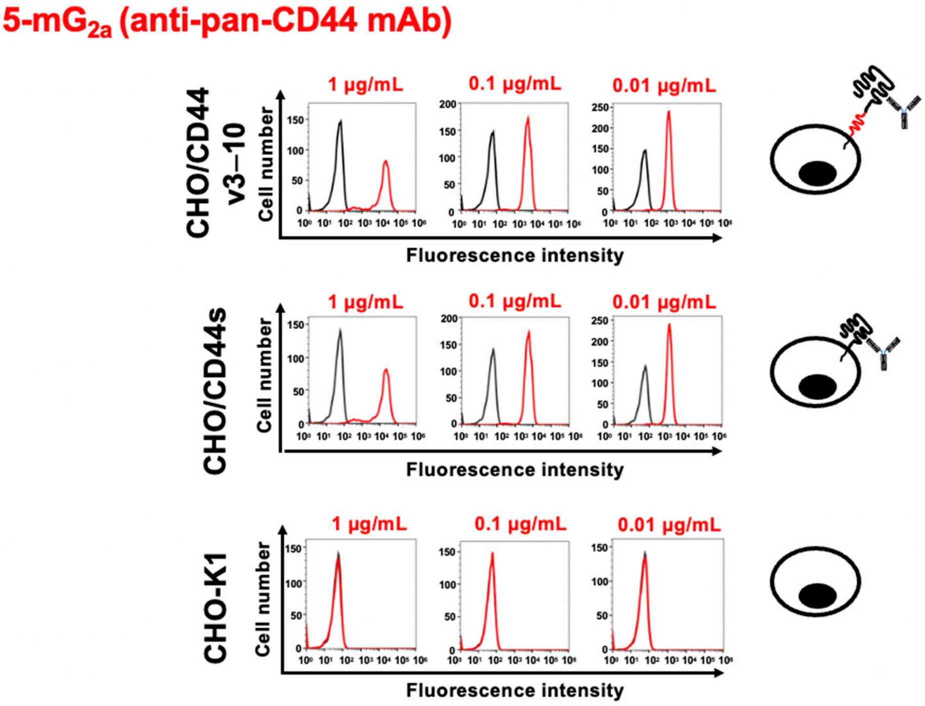

Supplementary Figure S1. Flow cytometry analysis using 5-mG$_{2a}$, an anti-pan-CD44 mAb. CHO/CD44v3–10, CHO/CD44s, and CHO-K1 were treated with 0.01, 0.1, and 1 µg/mL of 5-mG$_{2a}$. Then, the cells were treated with Alexa Fluor 488-conjugated anti-mouse IgG. Fluorescence data were analyzed using the SA3800 Cell Analyzer.

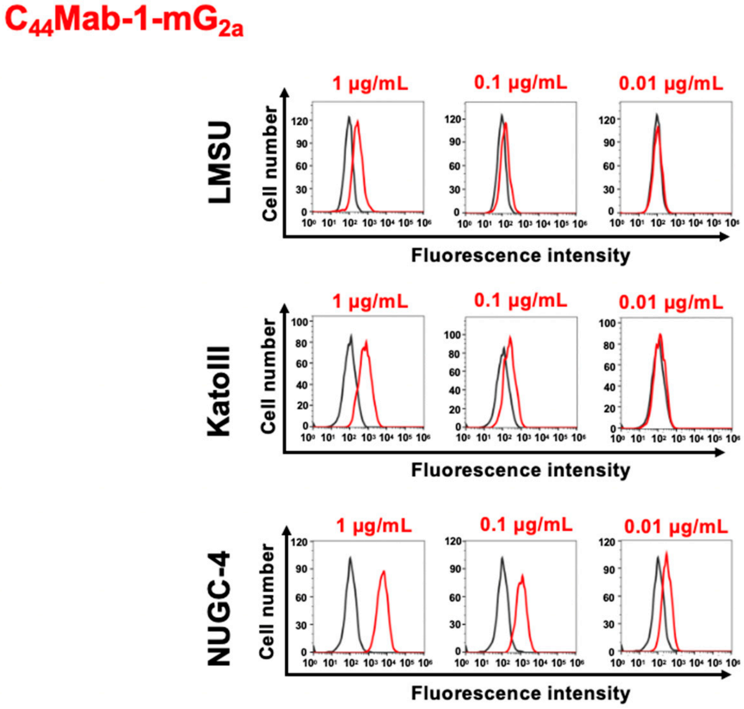

Supplementary Figure S2. Flow cytometry analysis of C_{44}Mab-1-mG_{2a} to CD44v9-positive gastric cancer cell lines. LMSU, KatoIII, and NUGC-4 were treated with 0.01, 0.1, and 1 μg/mL of C_{44}Mab-1-mG_{2a}. Then, the cells were treated with Alexa Fluor 488-conjugated anti-mouse IgG. Fluorescence data were analyzed using the SA3800 Cell Analyzer.

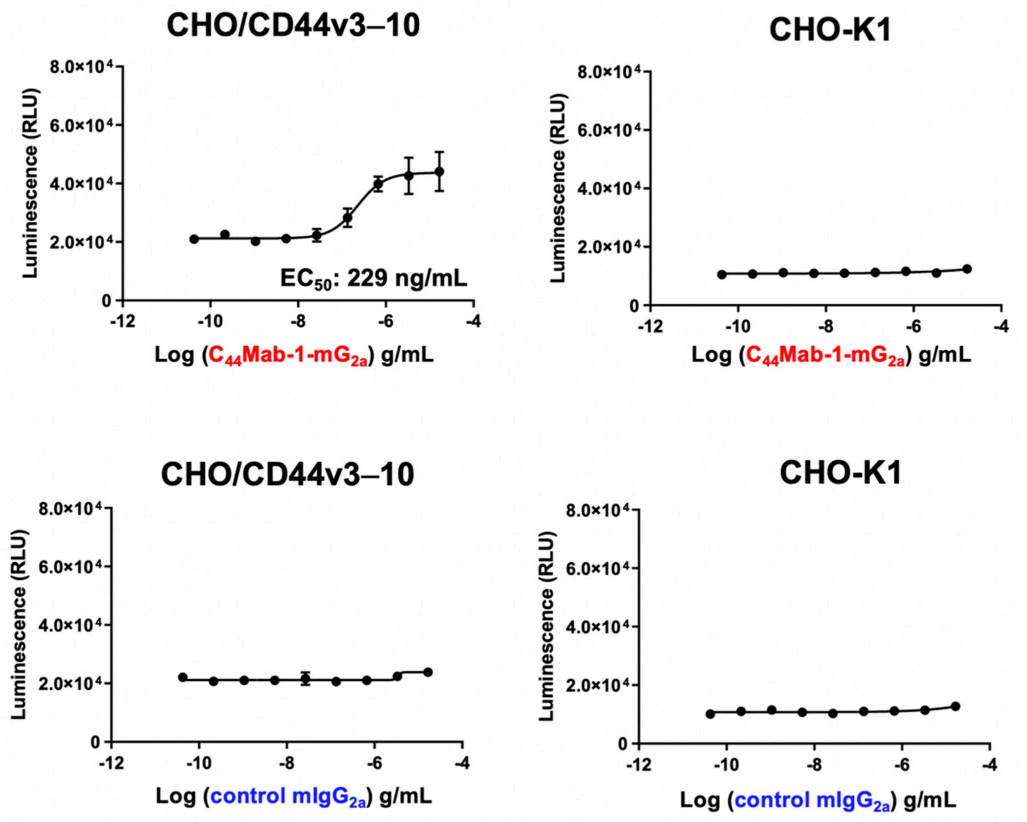

Supplementary Figure S3. The ADCC reporter assay by C_{44}Mab-1-mG_{2a} and PMab-231 (control mIgG_{2a}) in the presence of CHO-K1 and CHO/CD44v3–10 cells. The cells were cultured in a 96-well white solid plate. C_{44}Mab-1-mG_{2a} and PMab-231 were serially diluted and added to the target cells (n = 3). Jurkat cells stably expressing the human FcγRIIIa receptor and a NFAT-response element driving firefly luciferase were used as effector cells. The engineered Jurkat cells were added and co-cultured with antibody-treated target cells. Luminescence using the Bio-Glo Luciferase Assay System was measured using a GloMax luminometer (Promega Corporation, Madison, WI, USA). Values are presented as the mean ± SD. The EC_{50} was calculated using GraphPad PRISM 6.
