# Supplementary material for: Antitumor Activity by an Anti-CD44 Variant 9 Monoclonal Antibody in Gastric and Colorectal Cancer Xenograft Models
Source: Int J Mol Sci. 2025 Sep 19;26(18):9170. doi: 10.3390/ijms26189170 (PMC12470293; doi:10.3390/ijms26189170)
Supplement: Supplementary file 1 [file ijms-26-09170-s001.zip › Supplementary Table S1 C44Mab-1-mG2a-f, C44Mab-46-mG2a-f gastric cancer(BS01011b) .pdf]

Supplementary Table S1. Immunohistochemical analysis using C_{44}Mab-46-mG_{2a} and C_{44}Mab-1-mG_{2a} against gastric cancer tissue array (BS01011b).

No.	Age	Sex	Pathology diagnosis	TNM	Grade	Stage	C_{44}Mab-46-mG_{2a}		C_{44}Mab-1-mG_{2a}	
							tumor	stroma	tumor	stroma
1	55	F	Adenocarcinoma	T2N0M0	1	IB	+	+	+	-
2	51	F	Adenocarcinoma	T2N0M0	-	IB	+	-	-	-
3	71	M	Adenocarcinoma	T3N1M0	1	IIB	++	+	+++	-
4	63	M	Adenocarcinoma	T3N0M0	1	IIA	+	+	+	-
5	61	M	Adenocarcinoma	T2N0M0	1	IB	+	+	+	-
6	61	M	Adenocarcinoma	T2N0M0	1	IB	+	+	+	-
7	60	M	Adenocarcinoma	T3N2M0	1	IIIA	+	+	-	-
8	54	M	Adenocarcinoma	T3N2M0	1	IIIA	+++	+	+++	-
9	46	F	Adenocarcinoma	T3N0M0	1	IIA	+	+	+	-
10	66	M	Mucinous adenocarcinoma	T3N0M0	2--3	IIA	+	+	+	-
11	56	M	Adenocarcinoma	T2N0M0	2	IB	+	+	+	-
12	52	F	Adenocarcinoma	T3N0M0	2	IIA	++	+	+	-
13	70	M	Adenocarcinoma	T3N0M0	2	IIA	++	-	+	-
14	71	M	Adenocarcinoma	T2N0M0	2	IB	++	+	++	-
15	61	M	Adenocarcinoma	T3N0M0	2	IIA	-	+	-	-
16	75	M	Adenocarcinoma	T3N1M0	2	IIB	-	+	-	-
17	72	F	Adenocarcinoma	T3N0M0	2	IIA	+	+	-	-
18	60	M	Adenocarcinoma	T3N0M0	2	IIA	+	+	+	-
19	63	F	Adenocarcinoma	T3N0M0	2	IIA	-	+	+	-
20	69	M	Adenocarcinoma	T2N0M0	2	IB	-	+	+	-
21	54	F	Adenocarcinoma	T3N0M0	2	IIA	+	-	-	-
22	50	F	Adenocarcinoma	T3N0M0	3	IIA	-	+	-	-
23	64	M	Adenocarcinoma	T3N0M0	3	IIA	++	-	+	-
24	59	M	Adenocarcinoma	T2N0M0	2	IB	+++	-	+++	-
25	59	M	Adenocarcinoma	T2N0M0	2	IB	-	+	+	-
26	44	M	Adenocarcinoma	T3N0M0	2	IIA	-	+	+	-
27	76	M	Adenocarcinoma	T3N0M0	2	IIA	+	+	+	-
28	56	M	Adenocarcinoma	T3N0M0	2	IIA	+	+	+	-
29	56	M	Adenocarcinoma	T2N0M0	2	IB	++	+	+	-
30	58	M	Adenocarcinoma	T3N0M0	2	IIA	+	+	+	-

31	94	M	Adenocarcinoma	T2N0M0	2	IB	+	+	++	-
32	56	F	Adenocarcinoma	T2N0M0	3	IB	+	+	-	-
33	56	M	Adenocarcinoma	T4N1M0	3	IIIA	++	-	+	-
34	51	F	Adenocarcinoma	T3N0M0	2	IIA	+	+	+	-
35	67	M	Adenocarcinoma	T3N0M0	2	IIA	+	+	+	-
36	53	M	Adenocarcinoma	T3N0M0	2--3	IIA	-	+	+	-
37	48	F	Adenocarcinoma	T2N1M0	3	IIA	++	-	++	-
38	58	M	Adenocarcinoma	T2N0M0	2	IB	-	+	-	-
39	61	M	Adenocarcinoma	T2N0M0	3	IB	-	+	-	-
40	62	M	Adenocarcinoma	T2N0M0	3	IB	+	+	++	-
41	65	M	Adenocarcinoma	T2N0M0	3	IB	+	+	+	-
42	47	F	Adenocarcinoma	T3N1M0	3	IIB	+++	-	+++	-
43	65	M	Adenocarcinoma	T2N0M0	-	IB	+	-	-	-
44	52	F	Adenocarcinoma	T2N0M0	3	IB	-	+	-	-
45	72	M	Adenocarcinoma	T2N0M0	3	IB	+	-	+	-
46	68	F	Adenocarcinoma	T3N0M0	3	IIA	++	-	+	-
47	56	M	Adenocarcinoma	T3N0M0	3	IIA	++	-	++	-
48	59	M	Adenocarcinoma	T3N1M0	3	IIB	+	+	+	-
49	62	M	Adenocarcinoma	T3N1M0	3	IIB	-	-	-	-
50	60	M	Adenocarcinoma	T3N1M0	3	IIB	+++	-	+++	-
51	64	M	Adenocarcinoma	T2N0M0	3	IB	+	+	+	-
52	69	M	Adenocarcinoma	T2N0M0	3	IB	++	+	++	-
53	75	M	Adenocarcinoma	T2N0M0	3	IB	+++	-	+++	-
54	48	M	Adenocarcinoma	T2N0M0	3	IB	-	-	-	-
55	59	M	Adenocarcinoma	T2N0M0	3	IB	-	+	-	-
56	64	M	Adenocarcinoma	T3N0M0	3	IIA	+	-	++	-
57	55	M	Adenocarcinoma	T2N0M0	3	IB	++	-	++	-
58	58	M	Adenocarcinoma	T3N0M0	3	IIA	++	+	+++	-
59	64	M	Adenocarcinoma	T3N0M0	3	IIA	+	+	+	-
60	67	M	Adenocarcinoma	T3N1M0	3	IIB	+	+	+	-
61	49	M	Adenocarcinoma	T2N0M0	3	IB	+	+	-	-
62	35	M	Adenocarcinoma	T3N1M0	3	IIB	+	+	+	-
63	45	F	Adenocarcinoma	T4N0M1	3	IV	++	-	++	-
64	43	M	Adenocarcinoma	T2N0M0	3	IB	+	+	+	-
65	56	M	Adenocarcinoma	T2N0M0	3	IB	-	+	-	-

66	66	M	Adenocarcinoma	T2N0M0	3	IB	+	+	+	-
67	60	M	Adenocarcinoma	T3N0M0	3	IIA	+	-	-	-
68	74	M	Adenocarcinoma	T2N0M0	3	IB	++	+	++	-
69	58	M	Adenocarcinoma	T2N0M0	3	IB	+	-	-	-
70	68	M	Mucinous adenocarcinoma	T2N0M0	2	IB	++	+	+++	-
71	50	M	Mucinous adenocarcinoma	T3N0M0	3	IIA	-	-	-	-
72	51	M	Papillary adenocarcinoma	T2N0M0	2	IB	-	+	+	-

-, No stain; +, Weak intensity; ++, Moderate intensity; +++, Strong intensity.
